# A Novel Pre-Processing Technique for Original Feature Matrix of Electronic Nose Based on Supervised Locality Preserving Projections

**DOI:** 10.3390/s16071019

**Published:** 2016-06-30

**Authors:** Pengfei Jia, Tailai Huang, Li Wang, Shukai Duan, Jia Yan, Lidan Wang

**Affiliations:** College of Electronic and Information Engineering, Southwest University, Chongqing 400715, China; jiapengfei200609@126.com (P.J.); 18580465830@163.com (T.H.); wangyu2007@email.swu.edu.cn (L.W.); yanjia119@163.com (J.Y.); ldwang@swu.edu.cn (L.W.)

**Keywords:** sensor data, electronic nose, SLPP, data pre-processing, wound infection

## Abstract

An electronic nose (E-nose) consisting of 14 metal oxide gas sensors and one electronic chemical gas sensor has been constructed to identify four different classes of wound infection. However, the classification results of the E-nose are not ideal if the original feature matrix containing the maximum steady-state response value of sensors is processed by the classifier directly, so a novel pre-processing technique based on supervised locality preserving projections (SLPP) is proposed in this paper to process the original feature matrix before it is put into the classifier to improve the performance of the E-nose. SLPP is good at finding and keeping the nonlinear structure of data; furthermore, it can provide an explicit mapping expression which is unreachable by the traditional manifold learning methods. Additionally, some effective optimization methods are found by us to optimize the parameters of SLPP and the classifier. Experimental results prove that the classification accuracy of support vector machine (SVM combined with the data pre-processed by SLPP outperforms other considered methods. All results make it clear that SLPP has a better performance in processing the original feature matrix of the E-nose.

## 1. Introduction

An electronic nose (E-nose) is an expert system which is composed of an array of gas sensors as well as a corresponding artificial intelligence algorithm. The E-nose is effective in dealing with problems of odor analysis [1,2], and has already been introduced into many fields such as disease diagnosis [3,4,5] and food engineering [6,7]. The sensor array of the E-nose has a characteristic of cross-sensitivity, namely different units in the array will make responses when facing the same smell, which can effectively avoid the decision-making risk brought by the single sensor. The original feature matrix which is extracted from the response of sensors is redundant, and much key information which plays a crucial role in helping the E-nose make the right judgment is submerged.

Previous work has confirmed that the E-nose can be used to detect wounds of patients in labs and it is feasible for the E-nose to detect bacteria including the in the investigation of bacterial volatile organic compounds (VOCs) from cultures and also from swabs taken from wound-infected patients [8,9,10,11]. In fact, there are many kinds of pathogenic bacteria which can lead to wound infection. Therefore, the same sampling experiment on one pathogenic bacterium must be repeated many times to make the E-nose learn about one kind of wound infection “deeply”; meanwhile, the sampling experiments based on different infection types are also necessary in order to let the E-nose distinguish more kinds of wound pathogens. Additionally, based on the original feature matrix extracted from the response curves of sensors, the distance of points from different classes is larger than that of points from the same class, namely the data structure of the original feature matrix is nonlinear.

When this original feature matrix of wound infection data is put into the classifier directly, the classification accuracy of the E-nose is not ideal because of the redundancy of this matrix. So in order to capture more useful information which can be analyzed and to improve the classification accuracy of the E-nose as well, some methods must be applied to process the original feature matrix before it is put into the classifier.

Many seemingly complex systems described by high-dimensional data sets are in fact governed by a low number of parameters. The low-dimensional representation of such high-dimensional data sets not only leads to a more compact description of the data, but also enhances our understanding of the system [12]. Manifold learning is such a data processing method which can efficiently find the meaningful low-dimensional embedding from high-dimensional nonlinear data, and it processes the nonlinear data structure from the input matrix to a new matrix. In addition, the low-dimension output is very attractive to us, because this output will be processed by the classifier of the E-nose; if its dimension is low, then the computing complexity of the classifier is lower, and less computing and storage resources are needed. Many manifold learning methods have been proposed up to now [13,14], and a lot of work has been done to improve their performance [15,16,17]. Meanwhile, there are also many successful applications of manifold learning [18,19,20,21,22].

However, the traditional manifold learning cannot provide explicit mapping expression, which makes it unable to process the new sample points even if it can deal with the acquired data. Specifically, in our study, a trained E-nose will be used to predict the class label of unknown wound infection data, which means it is inevitable to encounter new data, and if the method employed by the E-nose cannot deal with these new points, it will make the E-nose incapable in practical application. Thus, supervised locality preserving projections (SLPP), a linear approximation of the nonlinear Laplacian eigenmaps, is applied. It not only shares many properties of the nonlinear techniques [23,24,25,26,27], but it also provides explicit mapping expression. As far as we know, SLPP has not been used in the field of the E-nose.

In this paper, we will use SLPP to process the original feature matrix and output a new matrix which can improve the classification accuracy of the E-nose. In Section 2, materials and experiments are demonstrated clearly. In addition, we will give the whole mathematical derivation of SLPP in Section 3. Then all considered pre-processing methods will be used to deal with the original feature matrix of wound infection data, and the classification results will be presented, analyzed and compared in Section 4. Finally, the conclusions of this paper are drawn in Section 5.

## 2. Materials and Experiments

### 2.1. Materials and Experimental Setup

**Figure 1:**
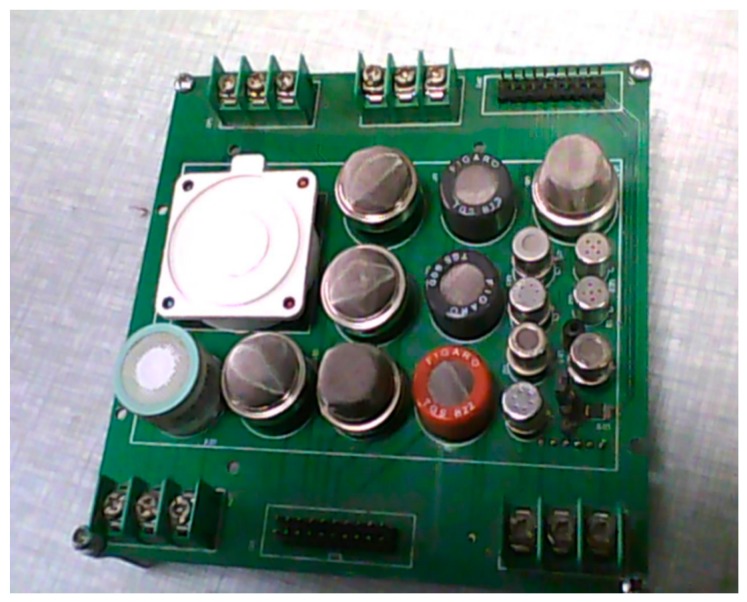
Electronic nose sensor array.

In this study, sprague-dawley (SD) rats are chosen as the targets of wound pathogen infection. There are four kinds of rats, uninfected and those infected with *S. aureus*, *E. coli*, and *P. aeruginosa*, respectively. Each rat has a wound in its right hind leg and the pathogens are inoculated in the wound. The metabolites in the reproduction process of the three pathogens are shown in Table 1. According to the metabolites of pathogens and the response characteristics of gas sensors, 14 metal oxide sensors and one electronic chemical sensor are adopted to construct the sensor array of this paper (shown in Figure 1). In addition, the sensitive characteristics of the 15 sensors are shown in Table 2.

**Figure 2:**
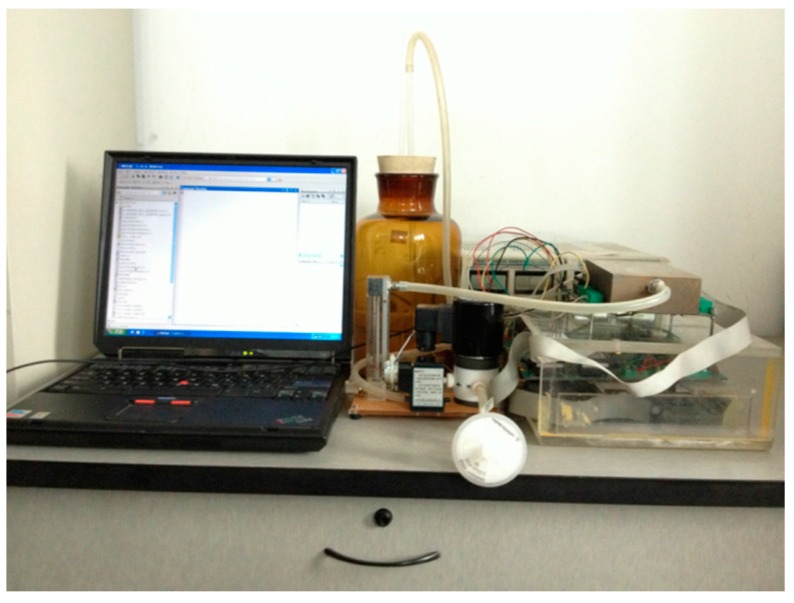
Practical E-nose system.

**Figure 3:**
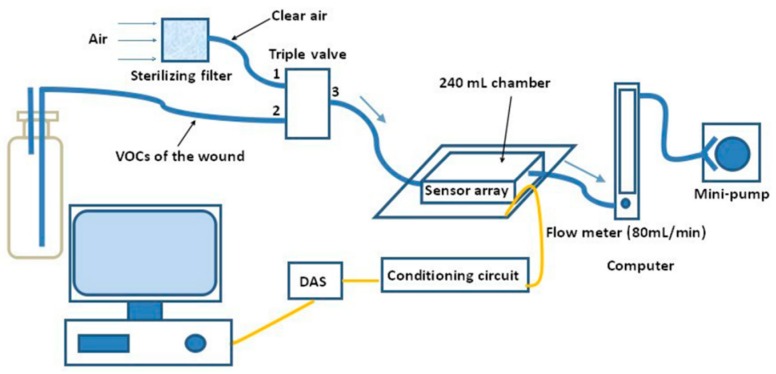
Schematic diagram of the experimental system.

The practical E-nose system is shown in Figure 2. The gas sensor array is placed in a stainless steel test chamber coated by Teflon with the volume of 240 mL. A triple valve is used to change the gas circuit to make sure the desired gas can flow into the chamber. The flow velocity of gas is controlled by a flow meter and a mini-pump, and its value is set as 80 mL/min. The response curves of the sensor array obtained from the wound odor of rats are firstly conditioned through a conditioning circuit and then sampled and saved in a computer via a 14-bit data acquisition system (DAS). A schematic diagram of the experimental system is shown in Figure 3.

### 2.2. Data Collection

Each rat is placed in a jar of which the volume is 2.8 L with a rubber stopper. There are two holes in the rubber stopper with two thin glass tubes inserted. One glass tube is fixed above the wound of the infected rat as closely as possible. The output gas of the tube which contains VOCs of the rat wound flows out of the bottle and then flows into the chamber through a Teflon tube.

The dynamic headspace method is adopted in all the sampling experiments. Each sampling experiment contains the following three steps:

Step 1: Set the triple valve to make port 1 connect to port 3, so clean air is exposed to the chamber and lasts for 3 min to wash the sensor array;

Step 2: Set the triple valve to make port 2 connect to port 3, so the gas stream containing VOCs of the wound passes over the sensor array for 5 min;

Step 3: Set the triple valve to make port 1 connect to port 3, so the sensors are exposed to clean air again for another 15 min.

**Figure 4:**
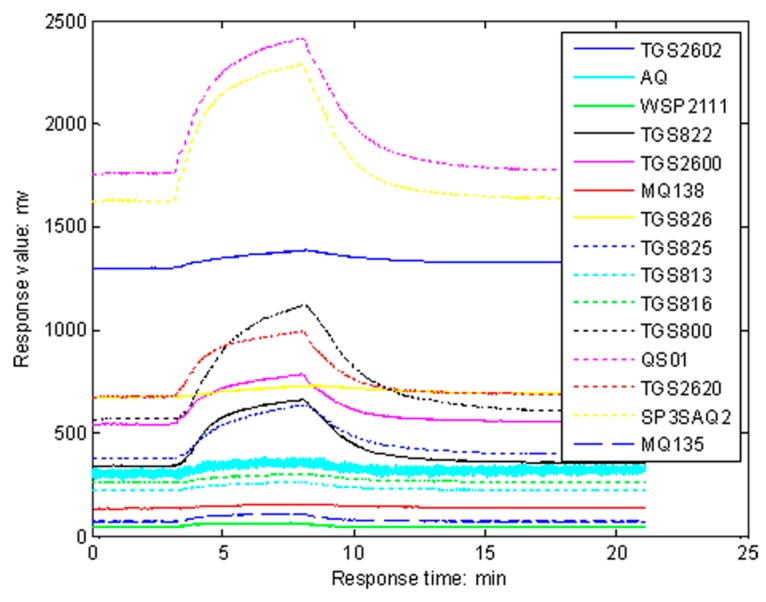
Response curves of 15 sensors on one wound infected with *P. aeruginosa*.

The clearance time between two experiments is 5 min. Twenty sampling experiments for each group of rats are made under the same conditions, and thus 80 sampling data sets can be collected. The response curves of 15 sensors on one wound infected with *P. aeruginosa* are shown in Figure 4. It can be seen that the obvious rise of each response curve is from the third min when the gas stream containing VOCs of the wound begins to pass over the sensor array, and the curves begin to drop from the eighth min when clean air is exposed to wash the sensor array.

### 2.3. Original Feature Matrix

**Figure 5:**
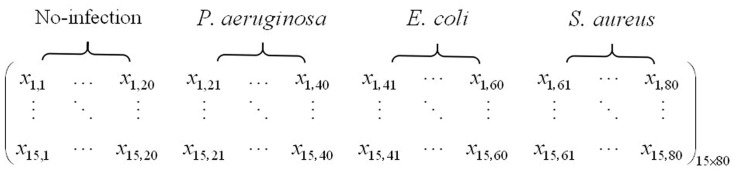
Detailed information of this original feature matrix.

After all the sampling experiments have been finished, we succeed in extracting the maximum steady-state response value of each sensor during each sampling experiment, and then the original feature matrix of the wound infection data is constructed. The detailed information of this original feature matrix is shown in Figure 5. As it shows, there are 80 points in this matrix and the dimension of each point is 15. Each class of wound infection contains 20 points.

To study the data structure of the original feature matrix, we use Equation (1) to compute the average Euclidean distance of points in the original feature matrix.

(1) $${\bar{d}}_{ij}=\frac{1}{M_{i}}\frac{1}{M_{j}}\sum_{p=1}^{M_{i}}\sum_{q=1}^{M_{j}}dis(x_{p},x_{q}),\;i,j=1,\;2,\cdots ,4$$

where ${\bar{d}}_{ij}$ is the average Euclidean distance between class *i* and *j*, *M_i_* and *M_j_* stand for the number of points in class *i* and *j*, and *dis (·)* is the Euclidean distance of points. The computed results are shown in Table 3. For uninfected wounds and those infected with *S. aureus*, the distance of points from the same class is less than that of the points from different classes. However, the distance between *E. coli* and no infection is less than that of the points between *E. coli* and other different classes, and the same situation happens in *P. aeruginosa*. This proves that the data structure of the original feature matrix is nonlinear and complex.

## 3. SLPP

Suppose there is a set of $x_{i}$, *i* = 1, 2, …, m in $R^{N}$, find a transformation matrix **A** mapping these points to a set of points $y_{i}$, *i* = 1, 2, …, m in $R^{L}$, such that $y_{i}$ will represent $x_{i}$, where $y_{i}=A^{T}x_{i}$. The algorithmic procedure of SLPP can be formally stated below.

(1) Constructing the neighborhood: $x_{j}$ becomes the neighbor of $x_{i}$ only if they are from the same class and are “close”, where both $x_{i}$ and $x_{j}$ are the points of **X** and i≠j. Additionally, two different ways can be employed to find the neighborhood of $x_{i}$.
  (a) ε-neighborhood: if ${\left\|x_{i}-x_{j}\right\|}^{2}<\varepsilon ，\varepsilon \in R$, then $x_{j}$ can be taken as the neighbor of $x_{i}$.
  (b) k-nearest-neighbors: a judgment is made on whether $x_{j}$ is among the k-nearest neighbors of $x_{i}$.
(2) Describe the relationship between $x_{i}$ and $x_{j}$: suppose that $w_{i,j}$ is a variable describing the relationship between these two points, and $w_{i,j}$ will be “larger” if $x_{i}$ and $x_{j}$ are “closer”. There are also two different methods available to realize it.
  (a) simple-type: $w_{i,j}=1$ if $x_{j}$ is the neighbor of $x_{i}$; otherwise, $w_{i,j}=0$.
  (b) heat-kernel: $\{\begin{array}{ll} w_{i,j}=\exp(-\frac{{\left\|x_{i}-x_{j}\right\|}^{2}}{t}) & \text{If }x_{j}\text{ is the neighbor of }x_{i}\;\\ 0 & \text{Otherwise} \end{array}$
(3) Find the map: to make the relationship between $y_{i}$ and $y_{j}$ similar to that between $x_{i}$ and $x_{j}$; let Y be a “good” map to minimize the following objective function [27].
  (2) $$F(y_{i},y_{j})={\sum_{i,j}\left(y_{i}-y_{j}\right)}^{2}w_{i,j}$$
  under appropriate constraints, where $y_{i}$ and $y_{j}$ are the points of Y and i≠j. If $x_{i}$ and $x_{j}$ are “close” enough, then the value of $w_{i,j}$ will be much “larger”, and to make sure Equation (2) reaches its minimum, $y_{i}$ and $y_{j}$ must be “close” as well. In this way, Equation (2) transfers the local structure from matrix **X** to **Y**. Furthermore, because $y_{i}=A^{T}x_{i}$, Equation (2) can be computed as
  (3) $$\begin{array}{l} \frac{1}{2}{\sum_{i,j}\left(y_{i}-y_{j}\right)}^{2}w_{i,j}=\frac{1}{2}{\sum_{i,j}\left(A^{T}x_{i}-A^{T}x_{j}\right)}^{2}w_{i,j}\\ =\sum_{i,j}A^{T}x_{i}D_{i,i}{x_{i}}^{T}A-\sum_{i,j}A^{T}x_{i}w_{i,j}{x_{i}}^{T}A=A^{T}X(D-W)X^{T}A=A^{T}XLX^{T}A \end{array}$$
  where $D_{i,i}=\sum_{i,j}w_{i,j}$. **A** constraint is imposed as follows [23]
  (4) $$Y^{T}DY=1\Rightarrow A^{T}XDX^{T}A=1$$

Finally, the minimization problem is reduced to find

(5) $$\underset{A^{T}XDX^{T}A=1}{arg\;\min}\;A^{T}XLX^{T}A$$

The transformation matrix **A** minimizing Equation (5) can be given by the minimum eigenvalue solution to the generalized eigenvalue problem

(6) $$XLX^{T}A=\lambda XDX^{T}A$$

Let the vectors $a_{1},a_{2},...,a_{L}$ be the solutions of Equation (6), and order them according to their eigenvalues, $\lambda_{1}>\lambda_{2}>...>\lambda_{L}$. Thus, the embedding is as follows

(7) $$Y=A^{T}X,A=(a_{1},a_{2},...,a_{L})$$

## 4. Results and Discussion

### 4.1. Experimental Results

To verify the effectiveness of SLPP, the original feature matrix of wound infection is processed by principal component analysis (PCA) [28], Fisher discriminant analysis (FDA) [29] and kernel FDA (KFDA) [30]. The original feature matrix (defined as matrix **X**) which was introduced in Section 2.3 will be processed by these four different data processing methods to create a new feature matrix which has been denoted as **Y**. Finally, matrix **Y** is put into the classifier as its input. For SLPP, the k-nearest-neighbors method is employed to find the neighborhood, and if the size of the neighborhood is different, the local data structure will be changed which will finally influence the classification results of the E-nose. To solve this problem, the grid-searching method is adopted to set the number of the nearest neighbors in SLPP. The heat-kernel method is used to describe the relationship of points; the value of *t* will influence the performance of the heat-kernel, and so quantum-behaved particle swarm optimization (QPSO) [31] is used to find the optimal value of *t*.

In this paper, we employ support vector machine (SVM) [32] and k-nearest-neighbor (KNN) [33] as the classifiers, and the parameters of SVM are optimized by QPSO, and the size of the neighborhood in KNN is searched by the grid-searching method. The cross-validation method is adopted to train and test SVM, and the folds of cross-validation in this paper are 10, 40 and 80. The numbers of particles and iterations in QPSO are set as 60 and 500. Every single data processing method is evaluated by its corresponding classification results.

Table 4, Table 5, Table 6, Table 7, Table 8 and Table 9 list the classification accuracy of SVM and KNN based on PCA, FDA, KFDA and SLPP when the folds of cross-validation are set as 10, 40 and 80, respectively. In addition, we also provide the classification results when matrix **X** is put into SVM directly without being processed by any method (no-dealing). It is evident, for the classification accuracy of the total four classes, that the best performance is achieved by SLPP, and the worst one is achieved by FDA, no matter if the fold of the cross-validation is 10, 40 or 80.

### 4.2. Discussion

This paper focuses on the investigation of the performance of SLPP in pre-processing the original feature matrix of wound infection data. Three other methods are also used to deal with this original feature matrix. When PCA is used to deal with the original feature matrix, its improvement is not obvious because PCA is good at finding and keeping the linear structure of data. FDA can make use of the class label information of the original feature matrix and find a linear transformation which can maximize the between-class scattering and minimize the within-class scattering to achieve a new feature matrix. However, the performance of FDA in predicting the class of wound infection is the worst among all the processing methods. As an enhanced technique of FDA, KFDA firstly maps the data to the high-dimension space, and then finds the same transformation as FDA, and its classification results of wound infection are better than that of FDA. However, SLPP manages to preserve the local structure of the data set through finding and keeping the neighbors of each point, and it can make use of the class label information during the course of finding the nearest neighbors. Further experimental results prove that the classification accuracy of the E-nose increases when SLPP is used to pre-process the original feature matrix of wound infection data, and meanwhile, it reduces the dimension of points from 15 to 7, which can greatly decrease the computational complexity of classifier.

## 5. Conclusions

Although the sensor array of the E-nose is good at cross-sensitivity, the original feature matrix extracted from the response curves of sensors is redundant; meanwhile, the data structure in this matrix is nonlinear. Traditional manifold learning methods are capable of solving the nonlinear problem, but they cannot provide an explicit mapping expression, which limits their application in the field of the E-nose. As a novel manifold learning technique, SLPP can efficiently find the meaningful low-dimensional embedding from high-dimensional nonlinear data, and it can process the nonlinear data structure from the input matrix to a new matrix; furthermore, the explicit mapping expression given by SLPP makes it possible for the E-nose to process the new sampling points. 

The experimental results of this paper have proved that the classification accuracy of SVM combined with SLPP is much higher than that of other considered methods. All in all, SLPP is an ideal technique for the E-nose to pre-process its original feature matrix of wound infection data and improve its classification accuracy.

## Figures and Tables

**Table 1:** Pathogens in wound infection and their metabolites.

Pathogens	Metabolites
*S. aureus*	Acetic acid, Aminoacetophenone, Ammonia, Ethanol, Formaldehyde, Isobutanol, Isopentyl acetate, Isopentanol, Methyl ketones, Trimethylamine, 1-Undecene, 2,5-Dimethylpyrazine isoamylamine, 2-Methylamine
*E. coli*	Acetaldehyde, Acetic acid, Aminoacetophenone, Butanediol, Decanol, Dimethyldisulfide, Dimethyltrisulfide, Dodecanol, Ethanol, Formaldehyde, Formic acid, Hydrogen sulfide, Indole, Lactic acid, Methanethiol, Methyl ketones, Octanol, Pentanols, Succinic acid, 1-Propanol
*P. aeruginosa*	Butanol, Dimethyldisulfide, Dimethyltrisulfide, Esters, Methyl ketones, Isobutanol, Isopentanol, Isopentyl acetate, Pyruvate, Sulphur compounds, Toluene, 1-Undecene, 2-Aminoacetophenone, 2-Butanone, 2-Heptanone, 2-Nonanone, 2-Undecanone

**Table 2:** Sensitive characteristic of gas sensors.

Sensors	Sensitive characteristic
TGS800	Methane, Carbon monoxide, Isobutane, Hydrogen, Ethanol
TGS813	Methane, Propane, Ethanol, Isobutane, Hydrogen, Carbon monoxide
TGS816	Combustible gases, Methane,Propane, Butane, Carbon monoxide, Hydrogen, Ethanol, Isobutane
TGS822	Organic solvent vapors, Methane, Carbon monoxide, Isobutane, n-Hexane, Benzene, Ethanol, Acetone
TGS825	Hydrogen sulfide
TGS826	Ammonia, Ethanol, Isobutane, Hydrogen
TGS2600	Gaseous air contaminants, Methane, Carbon monoxide, Isobutane, Ethanol, Hydrogen
TGS2602	VOCs, Odorous gases, Ammonia, Hydrogen sulfide, Toluene, Ethanol
TGS2620	Vapors of organic solvents, combustible gases, Methane, Carbon monoxide, Isobutane, Hydrogen, Ethanol
WSP2111	Benzene, Toluene, Ethanol, Hydrogen, Formaldehyde, Acetone
MQ135	Ammonia, Benzene series material, Acetone, Carbon monoxide, Ethanol, Smoke
MQ138	Alcohols, Aldehydes, Ketones, Aromatics
QS-01	VOCs, Hydrogen, Carbon monoxide, Metane, Isobutane, Etanol, Ammonia
SP3S-AQ2	VOCs, Methane, Isobutane, Carbon monoxide, Hydrogen, Ethanol
AQ	Carbon monoxide, Methanol, Ethanol, Isopropanol, Formaldehyde, Acetaldehyde, Sulfur dioxide, Hydrogen, Hydrogen sulfide, Phenol, Dimethyl ether, Ethylene

**Table 3:** Average Euclidean distance of points in matrix X.

	No-Infection	*P. aeruginosa*	*E. coli*	*S. aureus*
No-infection	1155.5567	1372.7781	1325.8864	1344.9724
*P. aeruginosa*	1372.7781	1461.6700	1488.3676	1499.6072
*E. coli*	1325.8864	1488.3676	1416.4451	1523.1622
*S. aureus*	1344.9724	1499.6072	1523.1622	1100.3343

**Table 4:** Classification results of 10-fold using different data processing methods (SVM).

Methods	*L*	Classification Accuracy (%)				
		No-Infection	*P. aeruginosa*	*E. coli*	*S. aureus*	Total
No-dealing	15	85	85	90	85	86.25
PCA	10	90	90	85	85	87.5
FDA	3	75	80	85	85	81.25
KFDA	3	90	95	95	95	93.75
SLPP	7	100	95	100	100	98.75

*L* is the dimension of matrix **Y**, and for the no-dealing method, *L* is the dimensionality of matrix **X**; Total means the classification accuracy of the classifier in predicting the class label of the total 80 points; No-dealing means the original feature matrix is put into the classifier directly.

**Table 5:** Classification results of 40-fold using different data processing methods (SVM).

Methods	*L*	Classification Accuracy (%)				
		No-Infection	*P. aeruginosa*	*E. coli*	*S. aureus*	Total
No-dealing	15	85	90	90	75	85
PCA	10	90	80	90	85	86.25
FDA	3	75	80	70	95	80
KFDA	3	90	95	90	95	92.5
SLPP	7	100	95	90	100	96.25

**Table 6:** Classification results of 80-fold using different processing methods (SVM).

Methods	*L*	Classification Accuracy (%)				
		No-Infection	*P. aeruginosa*	*E. coli*	*S. aureus*	Total
No-dealing	15	80	80	95	75	82.5
PCA	10	85	85	90	75	83.75
FDA	3	75	80	70	95	80
KFDA	3	85	85	90	90	87.5
SLPP	7	100	85	90	100	93.75

**Table 7:** Classification results of 10-fold using different data processing methods (KNN).

Methods	*L*	Classification Accuracy (%)				
		No-Infection	*P. aeruginosa*	*E. coli*	*S. aureus*	Total
No-dealing	15	85	80	80	85	82.5
PCA	11	90	85	75	85	83.75
FDA	3	85	80	75	85	81.25
KFDA	3	95	90	90	90	91.25
SLPP	8	100	90	90	100	95

**Table 8:** Classification results of 40-fold using different data processing methods (KNN).

Methods	*L*	Classification Accuracy (%)				
		No-Infection	*P. aeruginosa*	*E. coli*	*S. aureus*	Total
No-dealing	15	80	80	75	80	81.25
PCA	11	85	80	75	85	81.25
FDA	3	75	75	70	80	77.5
KFDA	3	90	90	85	90	88.75
SLPP	8	100	90	85	100	93.75

**Table 9:** Classification results of 80-fold using different processing methods (KNN).

Methods	*L*	Classification Accuracy (%)				
		No-Infection	*P. aeruginosa*	*E. coli*	*S. aureus*	Total
No-dealing	15	75	75	75	85	77.5
PCA	11	80	80	75	85	80
FDA	3	75	75	70	85	76.25
KFDA	3	85	90	85	90	87.5
SLPP	8	100	80	85	100	91.25
